# Supplementary material for: Katanin-Dependent Microtubule Ordering in Association with ABA Is Important for Root Hydrotropism
Source: Int J Mol Sci. 2022 Mar 31;23(7):3846. doi: 10.3390/ijms23073846 (PMC8999029; doi:10.3390/ijms23073846)
Supplement: Supplementary file 1 [file ijms-23-03846-s001.zip › ijms-1583490-supplementary.pdf]

International Journal of
Molecular Sciences

Supplementary materials

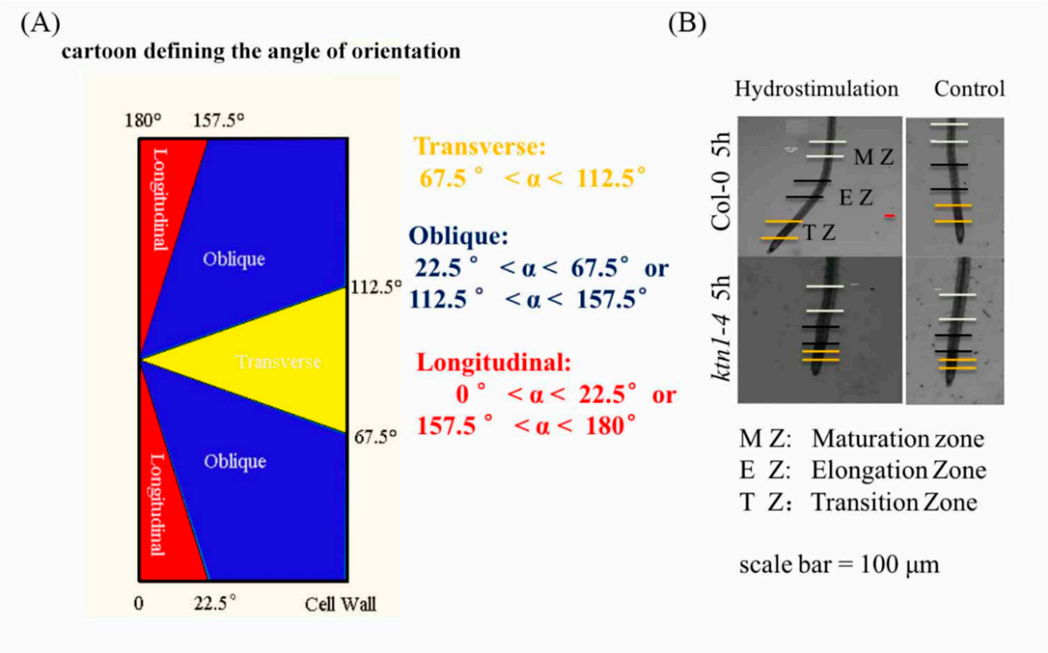

Figure S1. Cartoon representing the microtubule orientation angle (α). (**A**) Transverse: 112.5°≥α>67.5°. Oblique: 67.5°≥α>22.5° or 157.5°≥α>112.5°. Longitudinal: 22.5°≥α>0 or 180°≥α>157.5°. (**B**) Indicated maturation zone, elongation zone, and transition zone in WT and *ktn1-4* mutant roots before or after hydrostimulation treatment.

Table S1. Primers used in the study.

Name	Sequence
FBA2-F	CCTCGTCGAGCAGAACATTG
FBA2-R	TGTGAAATGGCAGCGTATCG
CLO4-F	CCAAACACGGAAGCGATTCA
CLO4-R	TGCAATACCTTCCACTCCGT
Actin-2-F	CCCGCTATGTATGTCGC
Actin-2-R	AAGGTCAAGACGGAGGAT
